# Fabrication of an electrospun polycaprolactone substrate for colorimetric bioassays

**DOI:** 10.1007/s10544-023-00673-z

**Published:** 2023-08-17

**Authors:** Chensong Xu, Gwenaël Bonfante, Jongho Park, Vincent Salles, Beomjoon Kim

**Affiliations:** 1 Department of Precision Engineering, School of Engineering, The University of Tokyo, Tokyo, 153-8505 Japan; 2 Institute of Industrial Science, The University of Tokyo, Tokyo, 153-8505 Japan; 3 LIMMS, CNRS-IIS UMI 2820, The University of Tokyo, Tokyo, 153-8505 Japan

**Keywords:** Glucose colorimetric sensing, Biosensing, Electrospinning, Microfiber sheet, Paper sensor, Polycaprolactone

## Abstract

Colorimetric assays rely on detecting colour changes to measure the concentration of target molecules. Paper substrates are commonly used for the detection of biomarkers due to their availability, porous structure, and capillarity. However, the morphological and mechanical properties of paper, such as fibre diameter, pore size, and tensile strength, cannot be easily tuned to meet the specific requirements of colorimetric sensors, including liquid capacity and reagent immobilisation. As an alternative to paper materials, biodegradable polymeric membranes made of electrospun polycaprolactone (PCL) fibres can provide various tunable properties related to fibre diameter and pore size.

We aimed to obtain a glucose sensor substrate for colorimetric sensing using electrospinning with PCL. A feeding solution was created by mixing PCL/chloroform and 3,3’,5’,5’-tetramethylbenzidine (TMB)/ethanol solutions. This solution was electrospun to fabricate a porous membrane composed of microfibres consist of PCL and TMB. The central area of the membrane was made hydrophilic through air plasma treatment, and it was subsequently functionalized with a solution containing glucose oxidase, horseradish peroxidase, and trehalose.

The sensing areas were evaluated by measuring colour changes in glucose solutions of varying concentrations. The oxidation reactions of glucose and TMB in sensor substrates were recorded and analysed to establish the correlation between different glucose concentrations and colour changes. For comparison, conventional paper substrates prepared with same parameters were evaluated alongside the electrospun PCL substrates. As a result, better immobilization of reagents and higher sensitivity of glucose were achieved with PCL substrates, indicating their potential usage as a new sensing substrate for bioassays.

## Introduction

Biosensing aims to detect the presence of biomarkers in the patients’ body (Promphet et al. 2020; Ray and Steckl 2019; Wang et al. 2021). It is an important tool in nowadays medical routine as an indicator of the patients’ body condition. A lot of different biofluids such as saliva, blood, sweat, tears, or interstitial fluid are used and each of them has advantages and drawbacks (Donati et al. 2020; Lee et al. 2020; Vyas et al. 2020; Zhang et al. 2016). They can help monitor the current condition as well as defining risk factors such as cancer, diabetes, etc. Each one has a specific composition in biomarkers but are all proportional (Corrie et al. 2015; Wang et al. 2021). Blood is the most widely used sample but involve invasive technics needing medical professional. Urine, sweat, or tear are linked to body response and not readily available. Furthermore, purification may be necessary. Among biofluids samples that are available currently, interstitial fluid (ISF), surrounding human cells, contains abundant plasma biomarkers with almost same composition as serum and plasma, making it a promising alternative to blood for biosensing (Miller et al. 2018).

Meanwhile, one of the most common targets in biosensing is glucose that is directly related to the diagnosis of diabetes. Diabetes is a chronic and metabolic disorder in glucose metabolism related to insulin secretion, which can seriously affect our body’s functioning. Along with diabetes, prediabetes is attracting enormous attention recently for the prevention of diabetes. Prediabetes corresponds to a blood sugar level higher than normal but still below the level to be diagnosed with diabetes type 2 (Bergman 2011; Tabák et al. 2012). This condition is based on impaired glucose tolerance (IGT) and impaired fasting glucose (IFG) in individuals. In the American population, it accounts for 20% of the 20 to 80 years old (Hostalek 2019). The reversible characteristic of prediabetes makes it very important to be detected the sooner since 80% of people suffering from it are not aware of it. Thus, it is very important to monitor and diagnose the glucose levels before prediabetes develops into diabetes.

Among several diagnostic techniques used for glucose diagnosis, electrochemical or colorimetric sensing methods are frequently used for its accuracy, availability, or usability. Electrochemical is based on the chemical oxidation or reduction of certain component into easily quantifiable and detectable molecules. Electrochemical sensing involves molecular changes through chemical redox reactions, causing electrical signal changes. When biomarkers are present in the medium, electrochemical reactions resulting in a current measurable with electrodes. From that current, amounts of biomarkers can be calculated and linked to potential pathologies. For example, when studying diabetes, previous literature demonstrated that when attaching gold nanoparticles to an electrode, glucose can be oxidized in glucose oxidase (Saei et al. 2013). This reaction releases peroxide hydrogen, a chemical product easily quantifiable. However, this method requires high cost, power to function and involve complex processes to be fabricated. On the other hand, colorimetric sensing is based on the detection of a colour change by the naked eye or image analysis. The detection can be done in liquid or solid phase, by deposition of the detection solution on a substrate. As one of the commonly used colorimetric sensing methods, TMB (3,3’,5,5’-tetramethylbenzidine) based colorimetric sensing relies on the colour change due to oxidation reactions of TMB. The liquid method used in this work is based on the production of hydrogen peroxide: an enzyme deposited on the substrate consumes the biomarker of interest, resulting in the production of hydrogen peroxide. This hydrogen peroxide reacts with a dye that leads to a reaction noticeable by the naked eye (Lee et al. 2020). Glucose oxidase converts glucose to gluconic acid, generating H_2_O_2_ as a byproduct (1); H_2_O_2_ oxidizes TMB in the presence of horseradish peroxidase as catalyst (2), resulting in a colour change from uncoloured to blue (λ_max_ = 370, 650 nm) (Bally and Gribnau 1989; Cattaneo and Luong 1994).

$$\begin{array}{c}Glucose+{O}_{2}\underrightarrow{Glucose oxidase}Gluconic acid+{H}_{2}{O}_{2}\#\left(1\right)\end{array}$$

$$\begin{array}{c}{\text{3,3}}^{{\prime }},{5}^{{\prime }},{5}^{{\prime }}-TMB+{H}_{2}{O}_{2}\underrightarrow{Horseradish peroxidase}{\text{3,3}}^{{\prime }},{5}^{{\prime }},{5}^{{\prime }}-TMB\left(ox\right)+{H}_{2}O\#\left(2\right)\end{array}$$

A correlation between the detected glucose concentration and the colour change of the chromogen could be established and used for colorimetric sensing of glucose. The most remarkable advantage of colorimetric sensing technology is its self-diagnostic feature, feasible at home without the help of trained medical staff allowing people to test themselves and seek help from professionals if needed. In addition, it also does not require power source and is easy to fabricate.

Regarding the substrate for the colorimetric assay, biodegradable and biocompatible materials are mainly used for safety as well as easiness of waste management. Those substrates include paper-based sensors and polymer-based materials such as polycaprolactone (PCL), alginate, chitosan, etc. and make self-diagnostic colorimetric test eco-friendly (Lee et al. 2020; Promphet et al. 2020). For the materials of a substrate, the paper is one of the most widely used one: it is cheap and easily available commercially (Apilux et al. 2020; Eaidkong et al. 2012; Gabriel et al. 2017). However, paper-based sensors, even if widely available and convenient, has some drawbacks: paper is sensitive to humid environment, easily damaged, and has limited morphologies and porosity. Indeed, tuning up the properties of paper fibres requires complex and time-consuming processes. On the other hand, biodegradable and biocompatible polymers can be a good alternative to the issues forementioned. Polymers can be shaped in multiple states: melted, spin-coated, dip-coated, electrospun, etc. (Ahmed et al. 2015; Lalia et al. 2013). Polymer for biosensing purposes includes the natural polymer as the substrate material for a sensing device to detect various compounds (Negm et al. 2020) and the hybrid polymer made by metal deposition for electrochemical sensing probe (Shukla et al. 2017).

Recently, electrospinning (ES) technique is attracting great attention to make fibre-based membranes as it can produce micro/nano fibres with well-controlled diameter and porosity. ES can fabricate the film made of micro/nano fibres by applying an electrical potential to charged droplets of polymeric solution. Parameters such as the voltage applied, the distance between the expulsing nozzle and the target or the solution feeding flow can be tuned to achieve a wide diversity of morphology. In addition, polymers can be chemically modified and tailored with additives ranging from simple carbon-black particles to complex species, such as enzymes, viruses, and bacteria (Greiner and Wendorff 2007). The electrospun fibres have been widely used in environment related sensing process. Regarding the heavy metal existence in water, mercury sensing could be carried out with electrospun fibres in colorimetric sensing method (Balusamy et al. 2019). Also, humidity sensing could be achieved with Zinc oxide (ZnO) based electrospun fibres (Horzum et al. 2011). To use electrospun fibres for sensing in the biomedical aspect is attracting increasing attention and cancer sensing devices based on electrospun fibres have been developed (Mane et al. 2020). Among polymers for biomedical applications, for example polylactide acid (PLA), polyurethane (PU), polyglycolic acid (PGA), polycaprolactone is one of the most widely used and studied biodegradable polymers (Jahani et al. 2015; Malagon-Romero et al. 2018). Combining electrospinning and polycaprolactone allow to precisely tune the morphology of the fabricated membrane regarding its porosity, thickness, elasticity, mechanical properties, etc. (Camposeo et al. 2013; Chou and Woodrow 2017; Rashid et al. 2021).

In this study, we developed a novel substrate for biosensing glucose by electrospinning PCL fibres. Firstly, we fabricated PCL fibres functionalized with TMB using ES technique. The characterization of fabricated PCL fibres with different experimental conditions was then performed. After adjusting the hydrophilicity and following functionalization, the newly developed sensing substrate was evaluated for reagent immobilization and sensing behaviour. In addition, the comparison with conventional paper substrates with same parameters were demonstrated and improved immobilization of chromogens as well as enhanced sensing behaviour was achieved.

## Materials and methods

### Materials

PCL (average molecular mass: 80,000 g/mol), chloroform (purity > 99%), ethanol (99.5%), glucose oxidase (from Aspergillus niger, 145,200 units/g solid), horseradish peroxidase (297,000 units/g solid, suitable for manufacturing of diagnostic kits and reagents), trehalose dihydrate (from Saccharomyces cerevisiae, > 99%,) and 3,3’,5’,5’-tetramethylbenzidine (purity > 99%) (TMB) were purchased from Merck KgaA, Corp. (Tokyo, Japan). D (+)-Glucose was purchased from Hayashi Pure Chemical Ind., Ltd. (Osaka, Japan). A phosphate buffer solution was purchased from Nacalai Tesque, Inc. (Kyoto, Japan). A high-voltage power supply (model HJPQ 30P1, Matsusada Precision, Japan) was used. The syringe pump was purchased from KD Scientific, Inc. (Holliston, MA) (model KDS 200).

A syringe of 5 mL with a metallic blunt tip of 18 gauge was used. Air plasma treatment was performed using a plasma generator (Sakigake YHS-R, Japan), and the water contact angle was measured using a solid-liquid interface analyser DropMaster 300 (Kyowa Interface Science Co., Ltd., Japan).

### Fabrication of PCL/TMB electrospun membrane

The ES feed solution was prepared by mixing the PCL/chloroform and TMB/ethanol solutions. First, PCL was dissolved in chloroform at a concentration of 10% (w/w) and stirred at 500 rpm overnight to obtain a homogeneous solution. In total, 1 mL of 150 mM TMB solution in ethanol was added to 19 mL of the previously prepared PCL solution, resulting in a final TMB concentration of 7.5 mM. Finally, 5 mL of the resulting solution was placed in a disposable syringe for ES. Was placed in a disposable syringe for ES.

**Fig. 1:**
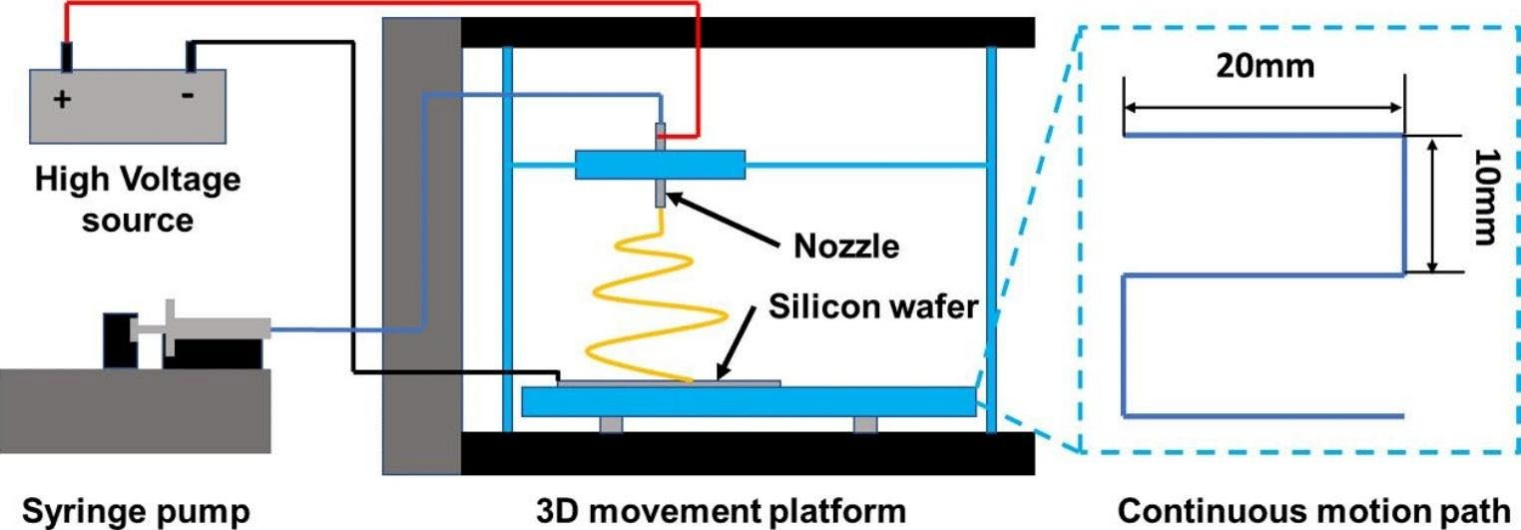
Set up for electrospinning with modified 3D printer

To achieve uniform deposition of electrospun PCL filaments on the collector, a custom ES setup was created using a modified 3D printer (Fig. 1). The original printing head of the printer was replaced with a flat-tip metal needle holder, and a silicon wafer was attached to the moving ground as the collector. The feeding solution in the syringe was then delivered to the flat-tip needle at a rate of 0.5 mL/h using a syringe pump and PTFE tube. A voltage of 8.8 kV was applied to the system at 15 cm between the needle tip and the collector. The ES process was continued for 90 min while the 3D movement platform repeating a continuous motion path consisted of three lines (20 mm in length) with pitches of 10 mm (Fig. 1) at a speed of 600 mm/min.

After the ES process was complete, the membrane was placed on the collector and stored in the dark overnight before being peeled off. Once the membrane was peeled off, its thickness was measured using a spiral micrometre. To evaluate the inner structures of the fabricated PCL/TMB membrane and conventional filter paper (Whatman® grade 4), scanning electron microscope (SEM) images were acquired using the following protocol. A 10 mm by 10 mm section was cut from the fabricated PCL membrane or filter paper. The cut samples were gold-coated using a Quick Coater (SC-701 MkII, Sanyu Electron Co., Ltd., Tokyo, Japan) and observed using SEM (JSM-6060, JEOL Ltd, Japan) at 10 kV for the PCL/TMB membrane and filter papers.

### Functionalization of PCL/TMB membrane

**Fig. 2:**
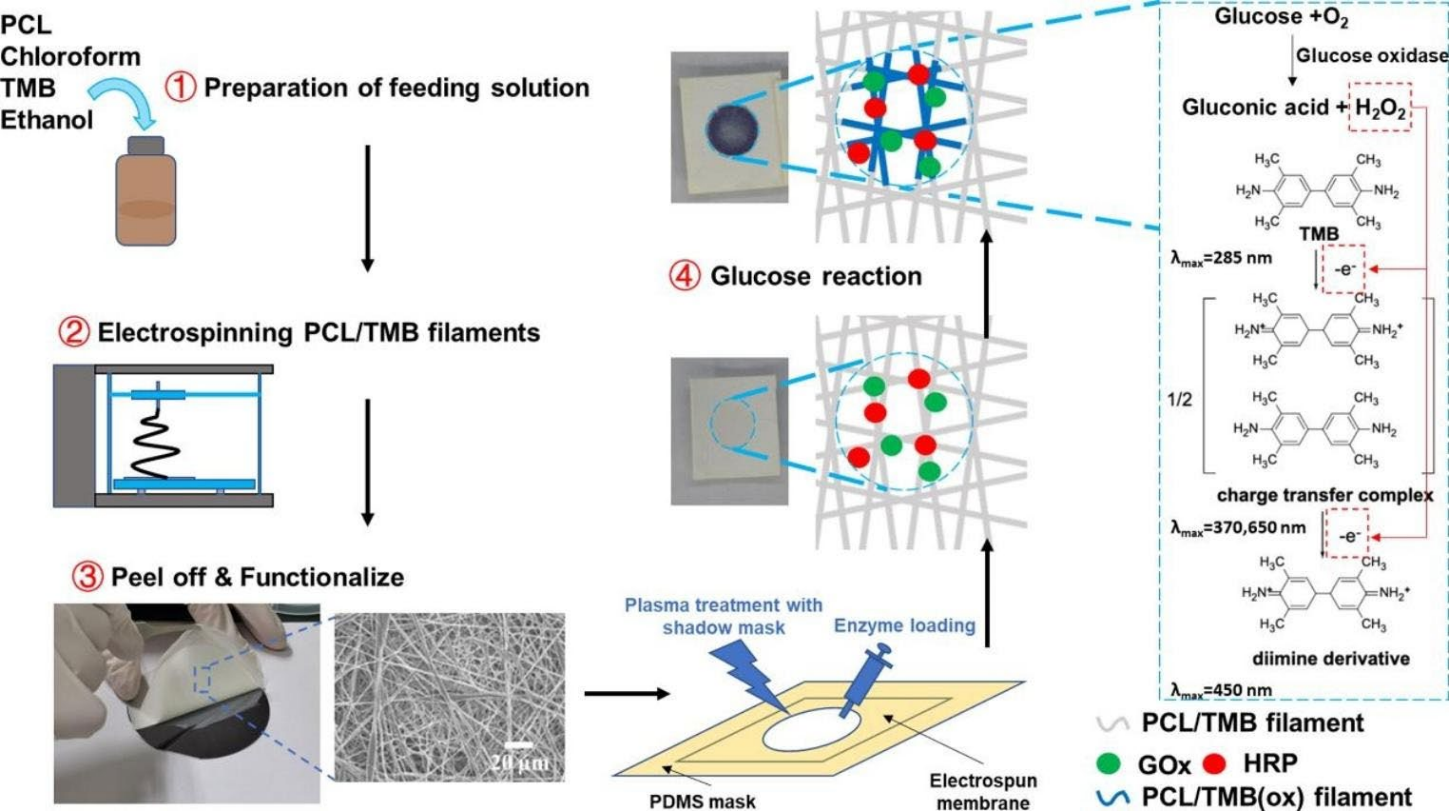
Fabrication & functionalization of PCL/TMB membrane and colorimetric reaction of TMB.

For glucose sensing, an enzymatic solution was prepared to contain 100 U/mL glucose oxidase, 100 U/mL horseradish peroxidase, and 250 mM trehalose in phosphate-buffered saline (PBS). To establish a designated hydrophilic area for the colorimetric assay, air plasma treatment was performed using a 5 mm thick Polydimethylsiloxane (PDMS) shadow mask with a 5 mm diameter hole at its centre. Following the plasma treatment, 4 µL of the enzyme solution was pipetted onto the hydrophilized area and left to dry completely in darkness at room temperature (26–28℃) for 1 h (Fig. 2). The red columns and arrows in Fig. 2 show the electron transfer in oxidation reaction of TMB.

### Evaluation of TMB immobilization

**Fig. 3:**
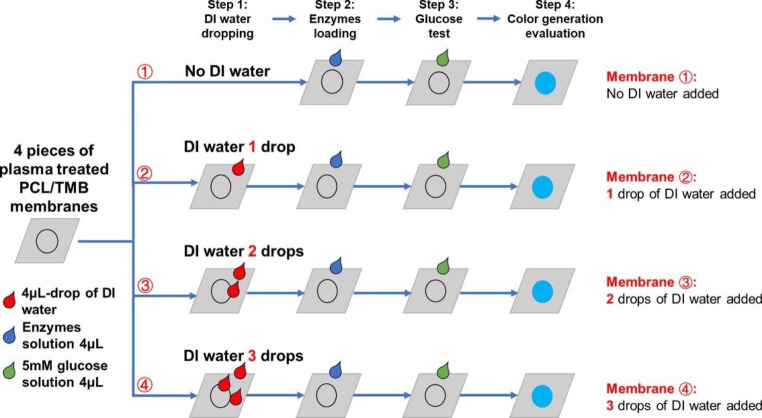
Schematics of TMB immobilization evaluation by dropping different quantity of DI water

In using the fabricated PCL membrane as a sensing substrate, uniform, and clear colour changes in colorimetric glucose sensing through proper immobilization of TMB must be ensured. To evaluate the immobilisation of TMB in the fabricated PCL/TMB membrane, DI water was used to mimic body fluids (Fig. 3). The hydrophilic area of the normally functionalized PCL/TMB membrane was expanded to a 9 mm diameter circular region to facilitate the flow process.

Before enzyme loading, 0 ~ 3 drops of Deionized (DI) water were pipetted onto four pieces of the PCL/TMB membrane. Subsequently, 4 µL of glucose solution at the same concentration were added to the membranes, and the resulting colour change was recorded using a camera. For comparison, the same procedure was performed on a paper substrate loaded with TMB. The results from the PCL/TMB and paper substrates were compared and analysed to evaluate the immobilisation of TMB.

### Evaluation of glucose sensing behaviour

Regarding the evaluation of the glucose sensing behaviour, we used the range of glucose concentration from 0 to 7 mM mainly as the glucose threshold for prediabetic state was suggested as 6.9 mmol/L by American Diabetes Association. First, the functionalized PCL/TMB membranes were subjected to glucose solutions ranging from 0 to 7 mM to assess the sensing areas. The oxidation reaction between glucose and TMB within the substrate resulted in a colour change that correlated with the glucose concentration.

After the colour was generated and gradually stabilised over 5 ~ 6 min, the colour change was recorded using a camera and analysed using ImageJ software. Colour images were converted to grayscale values to establish the correlation between glucose concentration and colour intensity on the sensing substrate. The change of greyscale value compared to that of 0mM glucose added membrane was calculated and plotted into a figure. The limit of detection (LoD) was calculated with the method introduced in (Holstein et al. 2015) while fitting the plots into 2-order equations instead due to limited concentration candidates. Whatman® grade 4 filter paper sensor substrates, with equivalent amounts of TMB and enzyme loadings, were fabricated and evaluated to compare with the PCL/TMB sensor substrates.

## Results and discussion

### Fabrication of PCL/TMB membrane

**Fig. 4:**
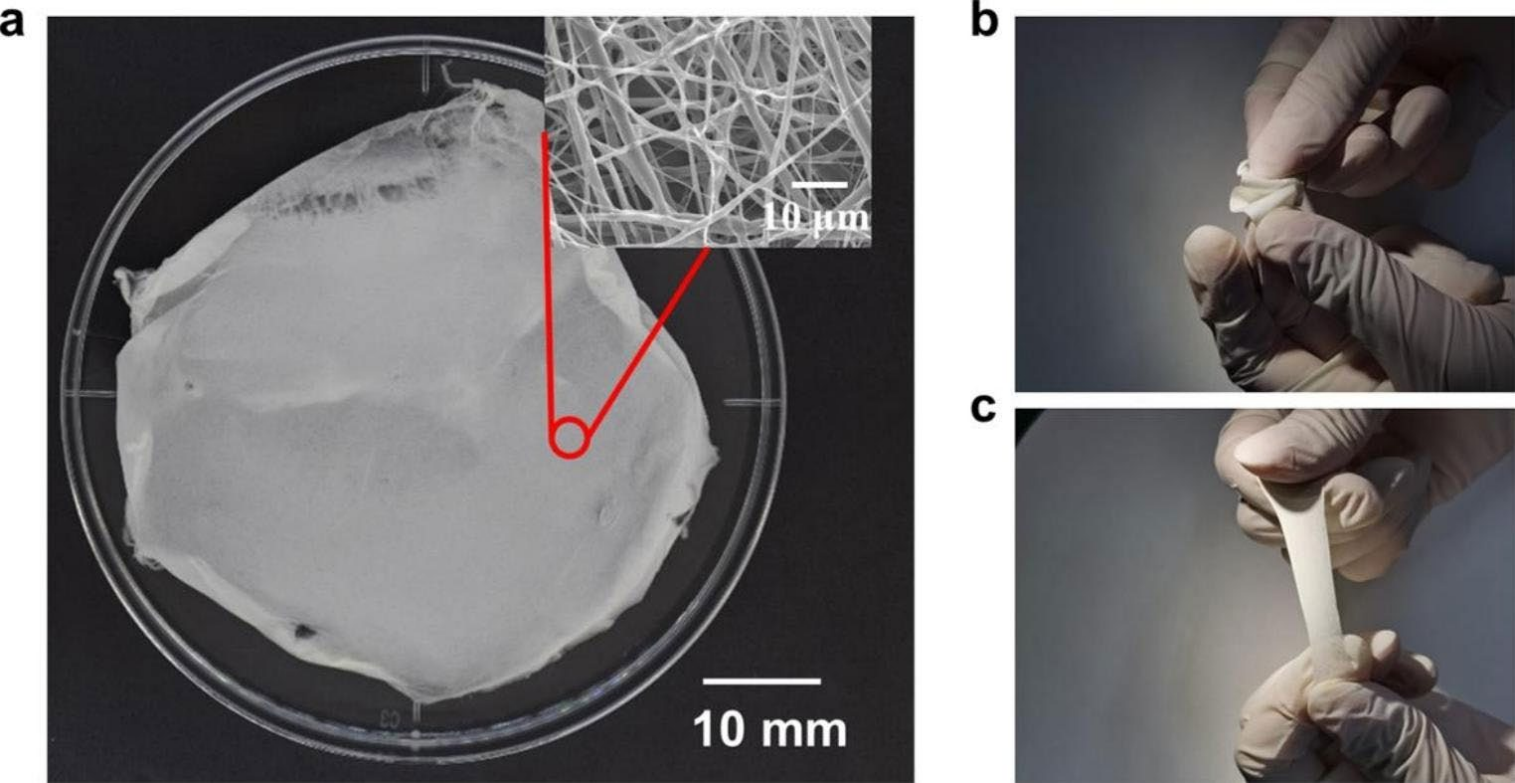
**(a)** Optical and SEM images of fabricated PCL/TMB membrane PCL/TMB membrane after peeling off **(b)** Flexibly folding of PCL/TMB membrane **(c)** Stretching of PCL/TMB membrane

The fabricated PCL/TMB membrane exhibited a flexible, white fabric-like appearance with a thickness of 250 ± 50 μm (Fig. 4a). Although TMB was integrated into the membrane and exposed to air and light during the ES process, the membrane remained white after fabrication, making it a suitable substrate for colorimetric sensing. Previous experiments with thinner membranes of 150 ± 50 μm thickness proved difficult to immobilize the TMB and give a satisfying colour development – coffee ring effect, inhomogeneous colour on the sensing area.

The fabricated membrane was sufficiently flexible to fit the skin surface without deformation from folding, making it suitable for use as a flexible sensing substrate that can be applied to human skin. According to references (F. Croisier et al., 2012) and (Kim 2008), the young’s modulus of electrospun PCL membrane ranges from several to several tens of million-pascals (MPa). Compared to the young’s modulus of Whatman filter paper series, which ranges from 0.46 to 1.71 GPa (Fernandes et al. 2017), the much lower young’s modulus of electrospun PCL membranes indicates a higher flexibility to fit human skin. Also, the bursting strength of electrospun PCL membranes was evaluated as several hundreds of kilopascals (S. Sharifi et al., 2022), while the one for the Whatman filter paper series can decrease to several kilopascals upon liquid absorption. The higher bursting strength of electrospun PCL membranes indicates a lower risk of breaking. After shape trimming and storage in darkness at room temperature, the fabricated PCL/TMB membrane sheets retained their original white colour for at least two weeks following fabrication.

**Fig. 5:**
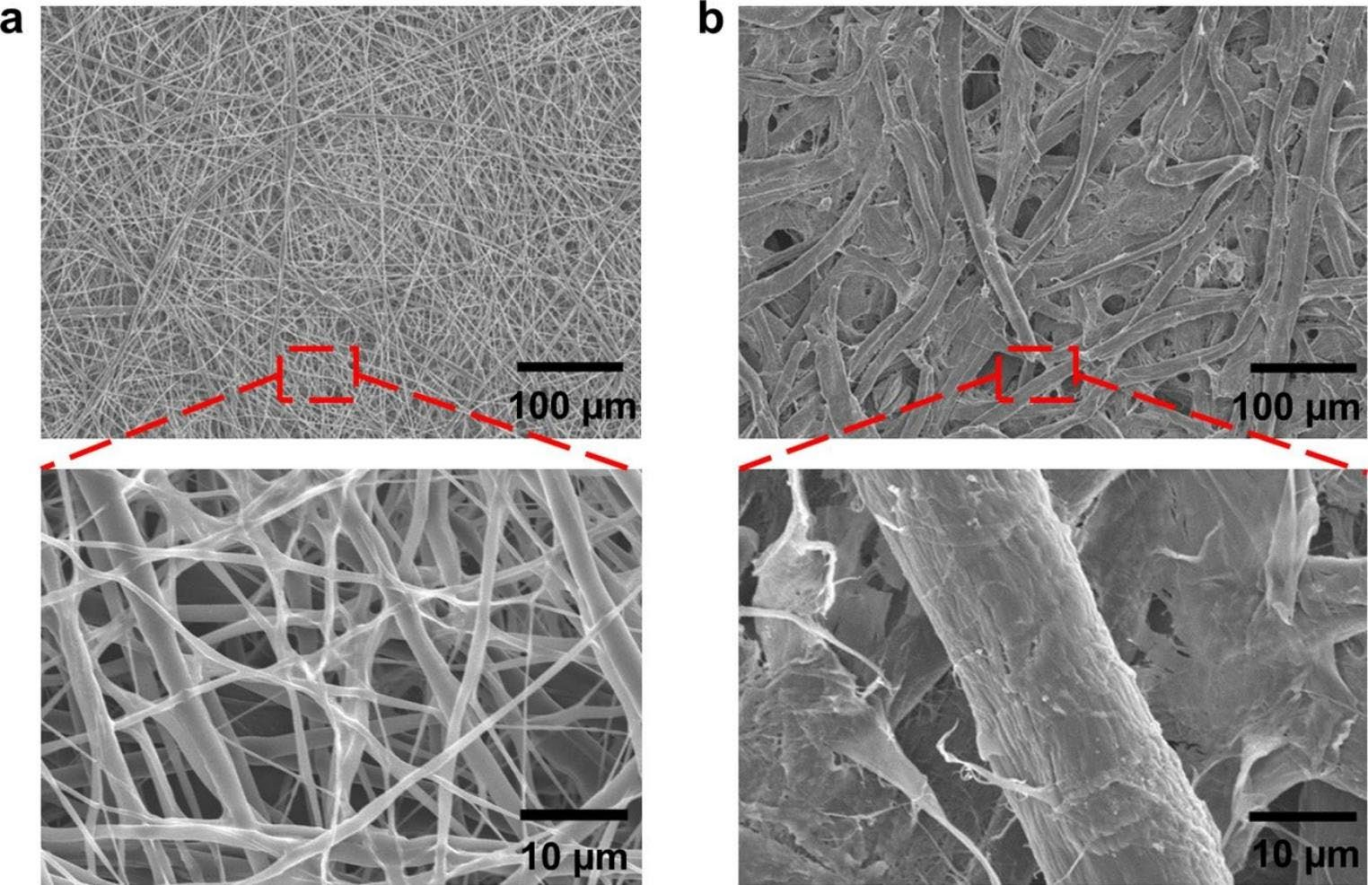
SEM image of fabricated PCL/TMB membrane **(a)** and filter paper **(b)**

The inner structure of the fabricated PCL/TMB membrane was observed and compared with that of Whatman® grade 4 filter paper (Fig. 5). The PCL/TMB membrane displayed a porous structure composed of microfibres that intertwined and covered each other. The diameter of microfibres within the PCL/TMB membrane was approximately 3.0 ± 1.6 μm. As the fabricated PCL/TMB membrane was a highly porous structure consisted of microfibers and voids totally interconnected, it is hard to define the item ‘pore size’ of this structure. To evaluate the size of voids, we introduced a measurement of void area. The voids were selected and fitted into best-fitting ellipse by using ImageJ software and the major/minor axis were measured. As a result, average major axis of 11.7 ± 3.8 μm and average minor axis of 7.0 ± 2.6 μm were obtained. In contrast, the porous structure of the filter paper exhibited fibre diameters ranging from 15 to 25 μm and an average major/minor axis of 6.5 ± 3.1/3.1 ± 1.0 μm.

Compared with the filter paper, the PCL/TMB membrane has thinner fibres and larger voids between the fibres, enabling the latter to achieve a larger surface area, which could be advantageous for colorimetric sensing (Doshi et al. 1995; Fong 1999; Norris et al. 2000). Surface area corresponds to the available surface of a sample and increases with the porosity of the porous structure. The larger cavities within the fabricated membrane not only enable a higher intake volume of fluid but also enhance sensitivity allowing the detection of molecules at low concentrations.

### Control over diameters of electrospun PCL/TMB filaments

The relationship between diameters of electrospun PCL/TMB filaments and three parameters in the electrospinning process (PCL weight ratio in feeding solution, working distance and flow rate) was evaluated from Tables 1 and 2.

Firstly, flow rate of feeding solution is fixed to be 0.5 mL/h, with working distance changing from 15 cm to 5 cm. Feeding solutions with 2 different PCL weight ratio (10% and 7%) were used in this process. As a result, the diameters of electrospun filaments from 7% (w/w) PCL feeding solution turn out to be smaller than those from 10% (w/w) PCL feeding solution for all three working distances. For electrospun filaments from same feeding solution, the diameters increase gradually as the working distance decreases. By applying different combinations of feeding solution and working distance showed in Table 1, average diameters of electrospun filaments ranging from 1.5 to 8 μm can be achieved.

**Table 1:** Diameters of electrospun PCL/TMB filaments with feeding solutions with different PCL weight ratios and different working distances

Working distance (cm)	Weight ratio of solution (w/w)	Fiber diameter (µm)
15	10%	4.01 ± 0.72
15	7%	1.93 ± 0.37
10	10%	5.71 ± 2.38
10	7%	2.62 ± 0.92
5	10%	7.89 ± 3.27
5	7%	7.82 ± 3.34

Secondly, the effect of flow rate on filament diameters is shown in Table 2. With PCL weight ratio of feeding solution and working distance being fixed as 10% and 15 cm respectively, the flow rate was changed from 0.5 to 0.1 mL/h. Diameters of electrospun filaments decrease as the flow rate decreases from 0.5 to 0.1 mL/h. When flow rate reaches 0.1 mL/h, filaments with an average diameter lower than 1 μm can be achieved while the finest filaments achieve diameters of less than 500 nm. The modification of diameters of electrospun filaments is the first step to modify the inner structure of whole porous membrane and the control over filament diameters shows the basic availability of achieving substrates with tunable morphology and mechanical characters.

**Table 2:** Diameters of electrospun filaments fabricated with different flow rates

Flow rate (mL/h)	Fiber diameter (µm)
0.5	4.01 ± 0.72
0.3	1.86 ± 0.61
0.1	0.97 ± 0.42

### Functionalization of PCL/TMB membrane

**Fig. 6:**
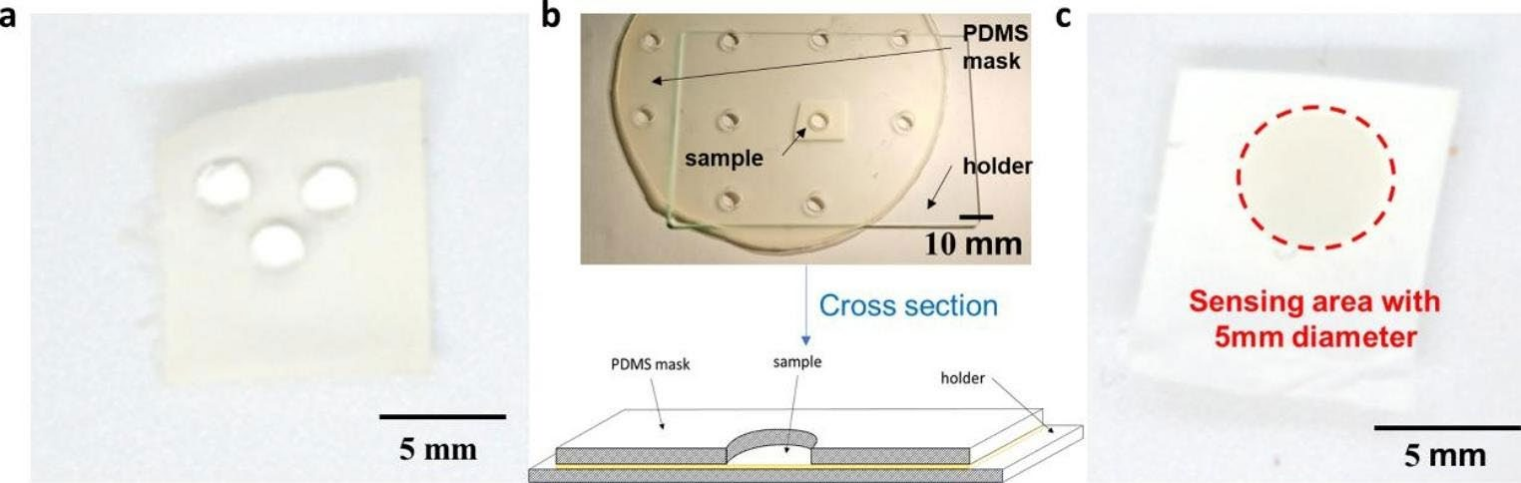
Hydrophilicity evaluation and shadow-masked plasma treatment schematic **(a)** PCL/TMB membrane before plasma treatment **(b)** Schematic and cross section of the PDMS masked plasma treatment set up **(c)** Constrained hydrophilicity sensing area on treated PCL/TMB membrane

The hydrophilicity of the fabricated PCL/TMB membranes was evaluated and adjusted to function as a sensing substrate. Initially, the fabricated membrane showed poor absorption of aqueous solutions due to the hydrophobic nature of the PCL material (contact angle of 120.2 ° with DI water, Fig. 6a). Figure 6a shows 3 droplets of DI water on hydrophobic surface of PLC/TMB membrane. To adjust the hydrophilicity of the PCL/TMB membrane, air plasma treatment was used, as it is a conventional and rapid method for modifying the wetting properties of materials (Jokinen et al. 2012). The plasma treatment time was set to 2 min to achieve complete hydrophilicity, and a PDMS shadow mask was used to delimit the hydrophilic sensing area (Fig. 6b).

After treatment, a circular sensing area with a 5 mm diameter at the centre of the fabricated membrane became hydrophilic, showing a contact angle of 0° when tested with DI water (Fig. 6c). Finally, the enzyme solution was successfully loaded onto the sensing area without surpassing its defined edge.

The functionalization process of PCL/TMB substrates is more complex compared to paper substrates due to the natural hydrophobic behaviour of the latter. Paper, being hydrophilic, allows for easy liquid extraction through capillary force but remains difficult to control the flow within a defined area only. However, the hydrophobic behaviour of PCL/TMB substrates is advantageous for their use as sensing substrates in case of the combination with wearable sensing patch, such as porous microneedle array patches (Bao et al. 2022; Lee et al. 2020). The shadow masked plasma treatment can accurately define the sensing area, simplifying image analysis and improving analysis results. Additionally, by adjusting the parameter setting of the plasma treatment, it is possible to control the flow rate of liquid within the porous structure of the substrate potentially modifying its sensing behaviour.

### Evaluation of TMB immobilization

**Fig. 7:**
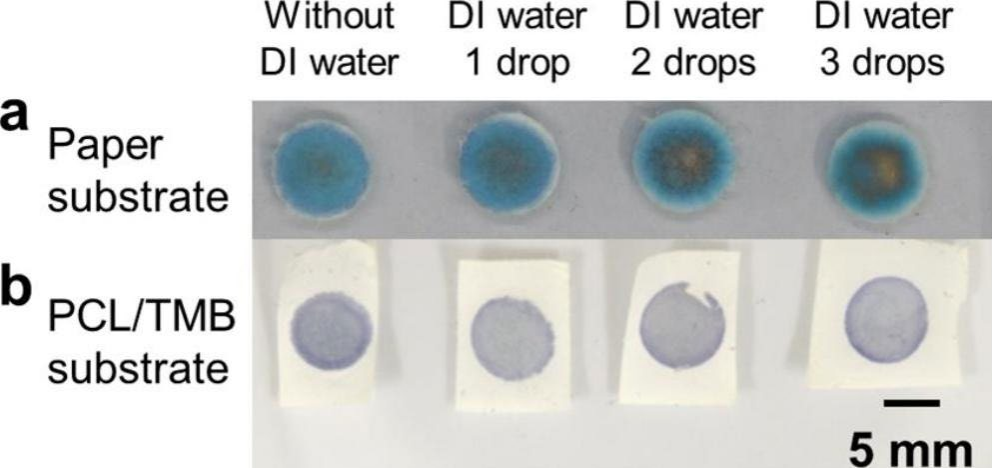
Results of evaluation of TMB immobilization **(a)** Evaluation result of paper substrate **(b)** Evaluation result of PCL/TMB substrate

The TMB immobilisation between the PCL/TMB membrane and paper substrates was compared (Fig. 7). Yellow coloration gradually appeared from the centre of the paper substrates, while the surrounding areas exhibited a blue hue as the drops of DI water were added. This colour change can be attributed to the two-step oxidation of TMB. The formation of a yellow diimine derivative indicates further oxidation of the blue charge transfer complex of TMB.

The generation of yellow colour from the centre part of the paper substrate could be explained by the insufficiency of TMB in the centre part due to the flow of TMB together with the liquid flow. TMB hardly dissolves in aqueous environments. However, TMB could be carried and flow together even with aqueous solutions if no immobilisation process was carried out. When some of the TMB originally deposited in the centre was carried towards the edge, the amount of TMB in the centre was not sufficient to consume all the H_2_O_2_ generated in the glucose oxidation reaction within the first step of TMB oxidation. Consequently, the blue complex was further oxidised into a diimine derivative, and a yellow over-oxidised area appeared in the centre. TMB was loaded onto the filter paper substrate by pipetting a TMB/methanol solution onto a trimmed piece of filter paper. As methanol evaporated, TMB remained inside the porous structure of the filter paper and was deposited on or between the fibres. In the paper substrates, the growth of the yellow area from the centre part and the shrinking of the blue area into the surrounding edge part indicate the travelling of TMB towards the edge part owing to poor immobilisation on the fibres of the porous structure. The fabricated PCL/TMB sensing substrate generated a uniform colour and showed no obvious difference as the number of drops of DI water increased, indicating better immobilisation of TMB on the microfibres of the porous structure.

### Evaluation of glucose sensing behaviour

**Fig. 8:**
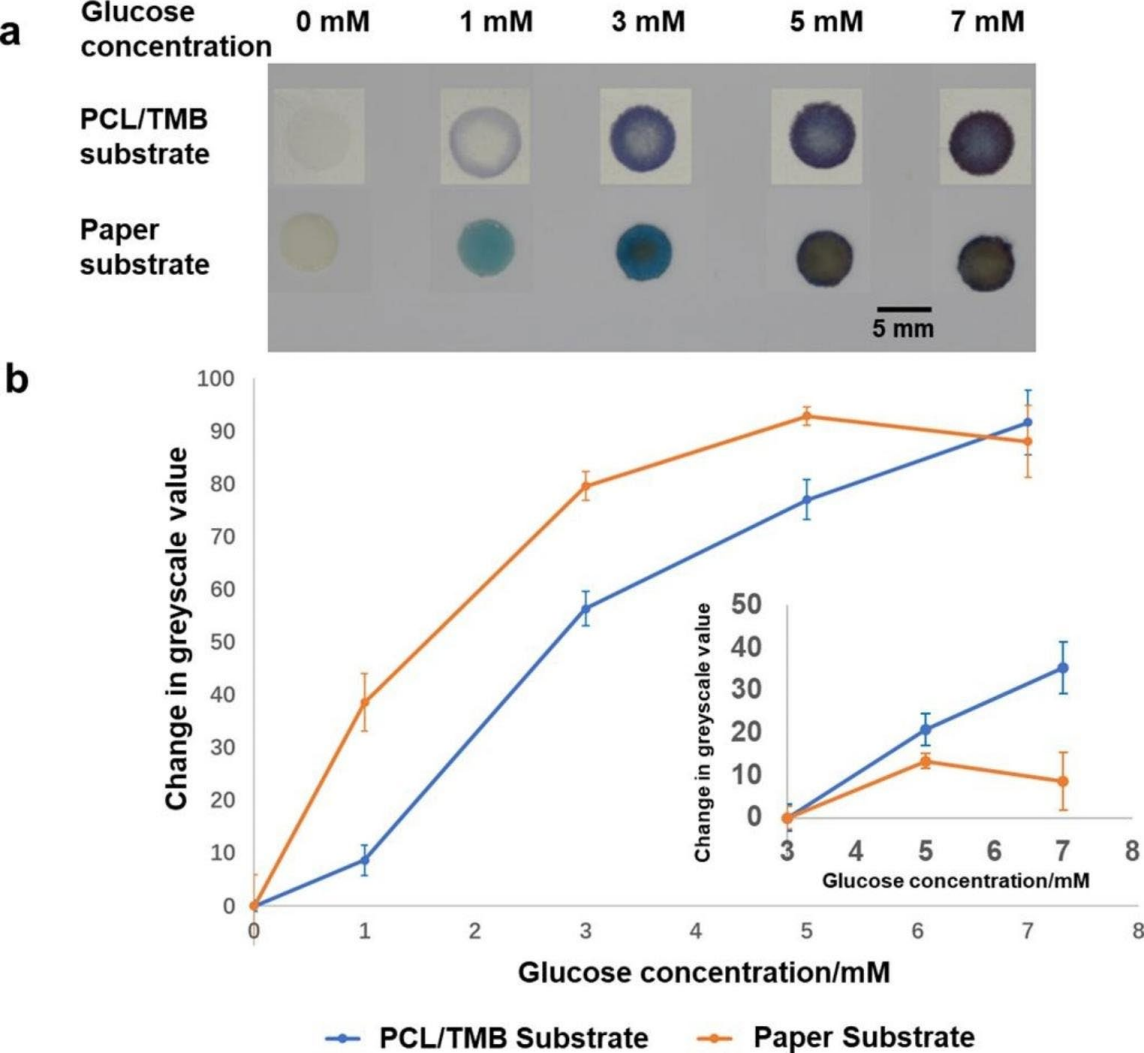
Comparison of colour change of PCL/TMB substrate and paper substrate on glucose sensing **(a)** Optical image of sensing behaviour of two kinds of substrates **(b)** Correlation between the change of greyscale value of sensing area and glucose concentration (with 0 mM as zero point)

The sensing of glucose solutions with concentrations of 0 ~ 7 mM was carried out (Fig. 8a). As the concentration of the glucose solution increased, the PCL/TMB sensing substrates displayed a uniform blue colour with gradually increasing intensity. Colour generation was uniform and well-constrained within the functionalized sensing area. The colour differentiation between the sensing areas was easily discernible to the naked eye. The colours were recorded with a camera and the greyscale values of coloured areas were obtained with ImageJ software. The greyscale value of the substrate loaded with 0 mM glucose solution (PBS solution only) was set as zero point. The differences between zero point and every greyscale value were calculated and plotted so that a correlation between the glucose concentration and the change of greyscale value was established (N = 4, Fig. 8b).

When the detected glucose concentration was increased by 1 or 2 mM, ranging from 0 to 7 mM, the change of grayscale value of the sensing area gradually increased from 0 to approximately 90. As the glucose concentration for pre-diabetes ranges from 3.9 to 6.9 mM, the detection range of 3 ~ 7 mM should be mainly paid attention to. The greyscale value of 3 mM glucose loaded substrate was set as zero point and the range of 3 ~ 7 mM was evaluated (Fig. 8b). For comparison, an identical process was performed using filter paper substrates (Fig. 8a, b).

An over-oxidised, yellow-coloured area began to form at a glucose concentration of 3 mM, intensifying with a higher concentration. When glucose concentration was over 3 mM, the sensing area of paper substrates were mainly occupied by the over-oxidised area, which made the colour distinction barely discernible. When glucose concentration increased above 5 mM, the change of greyscale value no longer performed a monotonically increasing trend, indicating the failure in this detection range. The overoxidation in the paper sensor substrates might be caused by insufficient TMB in specific areas. Electrospun PCL/TMB sensor substrates provided more uniform deposition and better immobilisation of TMB, as this was directly deposited with the PCL microfibres by ES rather than pipetted into a porous structure, as in paper sensor substrates. This advantage enables a higher sensitivity and wider detection range compared with paper sensor substrates with identical parameters. Indeed, the coated surface area was greater for the PCL membrane than the paper substrate. The limit of detection (LoD) of PCL sensing substrates was calculated according to the method introduced in (Holstein et al. 2015) with the evaluation results and LoD of 0.63 and 0.86 mM were obtained from PCL/TMB substrates and paper substrates respectively. The reason for the larger LoD compared to the former research (Lee et al. 2020) is the expansion of calculation range from 0 ~ 3 mM to 0 ~ 7 mM and the method used for image analysis. When glucose concentration rises to a high level, the standard deviations of greyscale value were found to increase, which influences the calculation of LoD. Simply representing the density of blue colour with greyscale value is not the most suitable method of image analysis and further improvement could be achieved by optimization.

Electrospun PCL/TMB sensor substrates fabricated in this study are expected to be used for various biomedical applications. As it is PCL polymer-based substrate, we expect it can be combined or fused to polymer-based sensing devices. For example, the PCL sensor substrate can be integrated with a polymer microneedle array patch (MAP) device. Microneedles (MNs) are an array of micro-sized needles arranged as a patch (Bao et al. 2022; Lee et al. 2020). Such needles, by their size, allow to reach the ISF by puncturing only the stratum corneum to reach the underlying epidermis (Gardeniers et al. 2003). In this epidermis, abundant ISF can be found, extracted, and analysed. Also, sensing substrates that can be properly combined with MNs patches are necessary for the integrity of MAP-based sensing system. With the integration of colorimetric sensing substrates on the back side of MNs patch, the ISF extracted by MAP will flow into the substrate with the help of capillary force. A colorimetric reaction will occur in the substrate. As a result, we expect that the realization of minimal-invasive MNs device for the daily diagnosis of pre-diabetes can be achieved by integrating MAP with the sensing substrate developed and optimized in our study.

## Conclusion

In this study, an electrospun PCL substrate for glucose sensing in colorimetric bioassays was fabricated and evaluated. By combining PCL/chloroform and TMB/ethanol solutions as the feeding solution for ES, we created a porous flexible membrane consisting of interwoven PCL/TMB microfibres.

ES was carried out at 8.8 kV with a feeding rate of 0.5 mL/h from a feeding solution containing 10% w/w PCL and 7.5 mM TMB. A modified 3D printer was used to ensure uniform deposition of the microfibres onto the collector. The working distance was set up at 15 cm. After 1.5 h, a porous PCL/TMB membrane with a thickness of 250 ± 50 μm was obtained, featuring microfibres with a diameter of around 3 μm and voids between them with diameters of approximately 10 μm.

Compared to filter paper substrates, commonly used in colorimetric sensing, the highly porous structure of the PCL/TMB substrate enables a larger surface area, contributing to enhancing sensitivity. The PCL/TMB membrane was shadow-masked and subjected to plasma treatment to create a well-constrained hydrophilic sensing area. After enzyme loading, the functionalized PCL/TMB membrane served as a sensing substrate for colorimetric glucose sensing.

TMB immobilisation was evaluated on the PCL/TMB and filter paper substrates using DI water to mimic the flow of ISF. The PCL/TMB substrate exhibited greater TMB immobilisation than the filter paper substrate because of the simultaneous ES of PCL and TMB from the same feeding solution. This ensured a more uniform and stable deposition of TMB molecules into the microfibres within the porous structure. Consequently, the PCL/TMB substrate demonstrated more stable and distinguishable colour generation in glucose colorimetric sensing compared to the filter paper substrate and the calculated LoD of PCL/TMB substrates also showed an advantage over that of paper substrates. This indicates a wider detection range and higher sensitivity in the biosensing of biomarkers. The use of electrospun membranes in colorimetric sensors presents a promising approach for developing wearable MAP sensors for colorimetric detection. Such membranes can be integrated into any existing fabric or MAP being developed, enabling the monitoring of biomarkers.
